# Role of Plasma Clusterin in Alzheimer’s Disease—A Pilot Study in a Tertiary Hospital in Northern India

**DOI:** 10.1371/journal.pone.0166369

**Published:** 2016-11-18

**Authors:** Venugopalan Y. Vishnu, Manish Modi, Sandeep Sharma, Manju Mohanty, Manoj Kumar Goyal, Vivek Lal, Niranjan Khandelwal, Bhagwant Rai Mittal, Sudesh Prabhakar

**Affiliations:** 1 Department of Neurology, Post Graduate Institute of Medical Education and Research, Chandigarh, India; 2 Department of Neurosurgery, Post Graduate Institute of Medical Education and Research, Chandigarh, 160012, India; 3 Department of Radiology, Post Graduate Institute of Medical Education and Research, Chandigarh, 160012, India; 4 Department of Nuclear Medicine, Post Graduate Institute of Medical Education and Research, Chandigarh, 160012, India; Florey Institute of Neuroscience and Mental Health, AUSTRALIA

## Abstract

**Objective:**

To evaluate the role of plasma clusterin in Alzheimer’s disease (AD).

**Background:**

Plasma clusterin is a promising biomarker as various studies have shown it to be associated with AD. But other studies have shown that plasma clusterin levels were not related to Alzheimer’s disease or presymptomatic AD. Hence the diagnostic value of plasma clusterin is still not conclusive.

**Methods:**

Neuropsychological assessment, MRI brain, FDG-PET brain and CSF biomarkers of AD were used for establishing the diagnosis of MCI, AD or Vascular dementia. The CSF control group included patients who were having knee or hip surgery and plasma control group included the spouses of patients.

**Results:**

Forty-six patients who gave consent for CSF examination and FDG PET brain were included in the study along with 19 control samples. Alzheimer’s group had 34 patients and Vascular group had 12 patients. Both had a significantly lower value of clusterin than the control samples (p<0.01). The median plasma clusterin level was 84.38 μg/ml in control group, 57.98μg/ml in Alzheimer’s group and 49.93μg/ml in the vascular group. Alzheimer and Vascular group did not differ in plasma clusterin levels. Moreover there was no correlation of plasma clusterin with AD severity. The sensitivity and specificity of plasma clusterin was low for any significance for clinical use.

**Conclusion:**

Our pilot study shows that plasma clusterin is lower in Alzheimer’s disease with respect to control population. Plasma clusterin levels and severity of Alzheimer’s disease had no significant correlation. There was no difference in plasma clusterin between Alzheimer’s disease and Vascular Dementia. The sensitivity and specificity of plasma clusterin is low for any use in clinical practice. More studies are required to ascertain the utility of plasma clusterin as a biomarker in Alzheimer’s disease.

## Introduction

Dementia is one of the biggest public health challenges the world is facing today. It has been estimated that 44.35 million people are living with dementia worldwide, which is projected to increase to 75.62 million by 2030 and 135.46 million by 2050.[1] The need for a reliable plasma biomarker in dementia, especially Alzheimer’s disease (AD) has led to many studies for evaluation of potential candidate biomarkers.

Of the various plasma biomarkers investigated for AD, clusterin has been one that holds promise. Clusterin gene (CLU) or Apolipoprotein J (Apo J) is the third most associated LOAD (Late Onset Alzheimer's Disease) risk gene according to Alzgene database.[2] In AD, various studies have shown elevated clusterin levels in brain and CSF.[3,4] Elevated plasma clusterin levels have been associated with brain atrophy, severity and progression of disease.[5,6] However, there are other studies that have shown no difference in plasma clusterin between dementia patients and controls suggesting that plasma clusterin levels may not be useful for diagnosis of AD.[5,7] Hence the role of plasma clusterin as a probable standalone biomarker in AD is still inconclusive.

## Methods

The study population was recruited from a tertiary care research institute in North India (Postgraduate Institute of Medical Education and Research, Chandigarh) and was approved by PGIMER institutional ethics committee. Written informed consent was taken either from the patient or from the spouse/first degree relative (if the patient was severely demented). The consent was recorded in a consent form (in both English and Hindi) which was approved by the Institute ethics committee. All investigations have been done according to the principles of Declaration of Helsinki. All consecutive dementia/Mild cognitive impairment patients attending neurology OPD were enrolled in the study.

Based on various diagnostic criterias (Dubois criteria for AD, DSM IV Criteria for VaD, National Institute on Aging- Alzheimer’s Association (NIA-AA) criteria for MCI) the patients were divided into the following subgroups: “Mild cognitive impairment-Alzheimer’s disease” (MCI-AD), “Mild cognitive impairment-Vascular” (MCI-VaSC), Alzheimer’s disease (AD), Vascular Dementia (VaD). The Alzheimer patients were subdivided based on the MMSE scores into mild (MMSE 21–26), moderate (MMSE 11–20) and severe AD (MMSE < = 10). MCI and Mild dementia were differentiated on the basis of involvement of activities of daily living. The Cerebrospinal fluid (CSF) control group included patients who were undergoing knee or hip surgery under spinal anesthesia and plasma control group included the healthy spouses of the patients after informed consent.

Assessment of the study population included clinical history, medical and neurological examination, laboratory investigation(Complete blood count, Electrolytes, Renal function tests, Liver function tests, HIV, VDRL, Vitamin B12), neuromaging {MRI Brain and Fluorodeoxyglucose Positron Emission Tomography (FDG PET)} and neuropsychological evaluation.

PET/CT imaging was performed using a Discovery LS (GE) PET scanner. Patients were fasting for at least 4 hours and blood glucose level <200mg/dl was ensured. Acquisition was initiated 45 minutes after intravenous administration of 10mCi (370MBq) of FDG. Reconstruction of data was done using iterative method (OSEM-Ordered subset expectation maximization method) and images were read in transaxial, coronal and sagittal views. The scans were evaluated qualitatively and visual analysis was used for the interpretation of the scans. The radiation burden to the persons due to the proposed study was minimal and well within the limits prescribed by atomic energy regulatory board (AERB) and there was no known harmful radiation effect from such procedures.

Neuropsychological assessment included MMSE, “Alzheimer’s Disease Assessment Scale (Cognitive)”, “PGI (Postgraduate Institute) Memory scale”, Verbal fluency–Controlled oral word test (phonemic) & Animal Names test (categorical), Quality of life–AD, “ADCS (Alzheimer’s Disease Cooperative Study)- Activities of Daily Living Inventory” and Hamilton depression rating scale.

Lumbar punctures were performed using a 22 gauge spinal needle without any blood contamination and 5 ml CSF was collected in polypropylene tubes, centrifuged and was analyzed for cell count, total protein, glucose, VDRL, ADA, Aβ42 and total tau (T-tau). CSF total tau and Aβ42 were determined quantitatively using a commercial sandwich enzyme linked immunosorbent assay (Invitrogen ELISA kits). ELISA analyses were performed according to the manufacturers’ protocols. Baseline venous blood samples were taken from patient and control group after overnight fasting. Serum Clusterin was measured in duplicate using commercial ELISA kit (Invitrogen ELISA kit). The minimum detectable dose of clusterin ranged from 0.064–1.050 ng/mL. Analyses were done at Experimental Pharmacology laboratory, PGIMER, Chandigarh, by operators who were blinded to all clinical information.

### Statistical analysis

ANOVA with multiple comparison using Bonferroni adjustment, Kruskal Wallis test with multiple comparisonand Mann Whitney U test were used for continuous ranked data. Categorical variables were analyzed using Chi square (Fisher’s Exact test). The difference in baseline characteristics (sex) was adjusted using multinomial logistic regression analysis. Data was analyzed using SPSS version 22.

## Results

A total of 222 patients were screened during the 16 months (January 2013 –April 2014). Of the 222 patients, 84 were excluded as per the exclusion criteria (Creutzfeldt Jakob disease -6,Tuberculous meningitis-10, Neurosyphilis- 1, HIV -6, Neurocysticercosis-5, Normal Pressure Hydrocephalus -6, Parkinson’s disease dementia-10,Traumatic brain injury-20, Frontotemporal dementia- 18, Primary progressive aphasia -1, Dementia with Lewy bodies-1).138 patients were initially included in the study. Twenty patients who did not come after the first visit were excluded.

**Fig 1:**
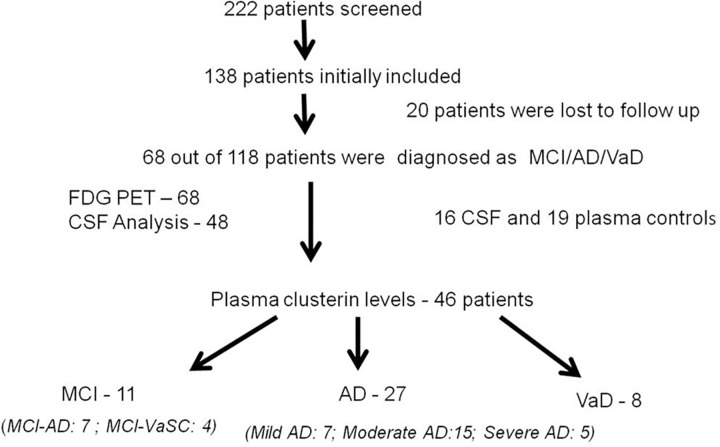
Flowchart Depicting the Study Design.

Of the remaining 118 patients, 68 patients who agreed to undergo either FDG PET or CSF analysis were included in the study. AD/MCI/VaD was diagnosed using clinical features, FDG PET or CSF biomarker profile {decreased Aβ42 and increased total tau (T-tau)}. Of the 68 patients, FDG PET was done in all while only 48 patients gave consent for performing Lumbar puncture. CSF biomarker analysis was done in these 48 patients. Plasma Clusterin levels were done in 46 of the total 68 patients (Fig 1). We recruited 16 healthy CSF controls and 19 healthy plasma controls.

Eleven patients were diagnosed with MCI, of which 7 were MCI-AD and 4 were MCI-VaSC. We had 27 patients with Alzheimer’s dementia (Mild AD- 7, Mod AD -15, Severe AD- 5) and 8 patients with vascular dementia. The Alzheimer pathology group (MCI-AD and AD) had 34 patients while Vascular group (MCI VaSC & VaD) consisted of 12 patients.

The median age of all the groups were comparable (Table 1). Gender was comparable among all the groups except in the plasma control group where there were more women. This is because the plasma group consisted mainly of asymptomatic spouses of the patients. Statistical analysis (multinomial logistic regression analysis) was done for adjusting the difference in sex. The educational status of patients was classified into three groups: Illiterate, School educated and College educated. All the groups were comparable at baseline regarding educational status. Various risk factors assessed at baseline (Hypertension, Diabetes, Coronary artery disease and Dyslipidemia) were comparable in all the groups.

**Table 1:** Baseline characteristics of the study and control population.

	MCI AD	MCI VaSC	AD	VaD	CSF CL	PL CL
Total patients	11	5	41	11	16	19
No of patients in whom clusterin was tested	7	4	27	8	-	19
Median age (yrs)	65	60	67	60	62	65
Inter-Quartile range	60–74	51.5–61.5	61.5–73.5	55–71	56.5–65	60–69
Male (No.)	9	4	24	10	10	5
Education- Illiterate	1	1	8	1	4	5
Education- school	5	3	27	9	10	10
Education college	5	1	6	1	2	4
HTN (No.)	8	4	10	10	8	8
DM (No.)	4	1	8	5	5	7
CAD (No.)	1	2	4	1	1	2
Dyslipidemia^a^ (No.)	2	4	4	8	8	12
Median duration of disease (months)	12	24	24	12		
MMSE (Median)	25	25	18	17	30	30

Abbreviations: AD = Alzheimer’ disease, CAD = coronary artery disease, CSFCL = CSF control, DM = diabetes mellitus, HTN = hypertension, MCI AD = mild cognitive impairment- alzheimers disease, MCI VaSC = mild cognitive impairment- vascular, MMSE = mini mental state examination, PLCL = plasma control, VaD = vascular dementia

^a^ Diagnosed according to NCEP guidelines

### Plasma Clusterin

Plasma Clusterin was measured in 46 patients and 19 controls. The median plasma clusterin level was 84.38 μg/ml in control group. It was 53.05 μg/ml in MCI-AD group, 61.32 μg/ml in AD group and 57.98 μg/ml in AD pathology group (Table 2). It was 53.27 μg/ml in MCI- VaSC group, 48.19 μg/ml in VaD group and 49.93 μg/ml in the vascular pathology group (Table 2).

**Table 2:** Plasma Clusterin levels in MCI, VaD, AD and control population.

	MCI AD (n = 7)	MCI VaSC (n = 4)	AD (n = 27)	VaD (n = 8)	Alzheimer group (n = 34)	Vascular group (n = 12)	Plasma control (n = 19
Clusterin median (μg/ml)	53.05	53.27	61.32	48.19	57.98	49.93	84.38
Clusterin Inter-Quartile Range	38.84–66.69	36.34–63.46	38.55–94.24	33.36–63.86	38.77–80.68	34.13–63.46	68.72–119.91

**Abbreviations**: AD = Alzheimer’ disease, MCI AD = mild cognitive impairment- alzheimers disease, MCI VaSC = mild cognitive impairment- vascular, VaD = vascular dementia

**Fig 2:**
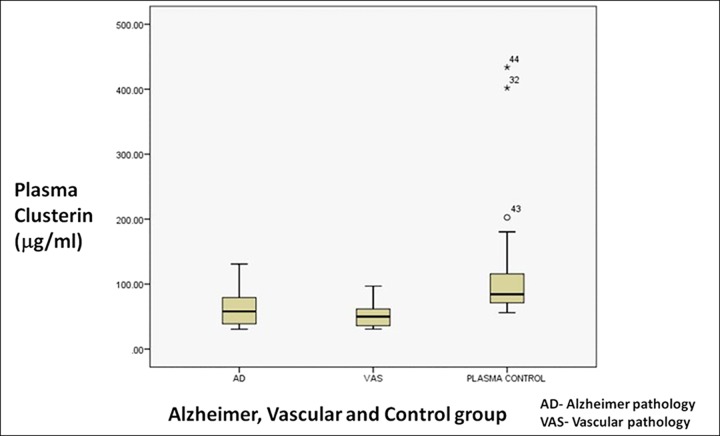
Box And Whisker Plot Depicting Plasma Clusterin Levels Of Alzheimer group, Vascular group And Control Population.

All the patient subgroups (MCI-AD, AD, Alzheimer pathology, MCI-VaSC, VaD and vascular pathology) had a significantly lower value of clusterin than the control samples (Fig 2). Among the subgroups there was no significant difference in the plasma clusterin levels. (Table 3, Fig 2). Similarly, among the AD severity subgroups also, there was no significant difference (Table 3). The alzheimer and vascular group had significantly lower clusterin value compared to control group even after adjusting for female sex which were more in the control group (Tables 4 and 5).

**Table 3:** Comparison of plasma clusterin among Alzheimer and vascular subgroups.

Parameter	Groups		p value
**Plasma clusterin**	MCI AD (n = 7)	MCI VaSC(n = 4)	0.850
	AD (n = 27)	VaD (n = 8)	0.188
	Alzheimer’s pathology (n = 34)	Vascular pathology (n = 12)	0.154
	Mild AD (n = 7)	Moderate AD (n = 15)	0.275
	Mild AD (n = 7)	Severe AD (n = 5)	0.167
	Moderate AD (n = 15)	Severe AD (n = 5)	0.359

**Abbreviations**: AD = Alzheimer’ disease, MCI AD = mild cognitive impairment- alzheimers disease, MCI VaSC = mild cognitive impairment- vascular, VaD = vascular dementia

**Table 4:** Comparison of mean clusterin among study groups using ANOVA with multiple comparison using Bonferroni adjustment.

Groups	N	Mean	SD	F value (df), pvalue	Multiple comparison using Bonferroni adjustment		
					Group comparison		p value
Alzheimer	34	65.44	28.32	8.217 (2.62) 0.001	Alzheimer	Vascular	1.000
Vascular	12	51.77	18.92		Alzheimer	Control	0.002
Control	19	130.18	108.66		Vascular	Control	0.004

**Table 5:** Multinomial logistic regression analysis to find out association between diagnosis groups and clusterin after adjusting sex of the patients.

Reference: control		beta	Standard error of beta	p value	Odds ratio	95% confidence interval Odds Ratio	
						Lower bound	Upper bound
Alzheimer	Intercept	3.099	0.968	0.001			
	Clusterin	-0.025	0.011	0.026	0.975	0.954	0.997
	Female	-0.753	0.688	0.274	0.471	0.122	1.814
	Reference: Male						
Vascular	Intercept	3.596	1.294	0.005			
	Clusterin	-0.047	0.019	0.015	0.954	0.918	0.991
	Female	-1.727	1.007	0.086	0.178	0.025	1.279
	Reference: Male						

Using ROC (Receiver operating characteristic) curve analysis, a plasma clusterin value of 80.8μg/mL had a sensitivity of 76.5% and specificity of 63.2% in differentiating Alzheimer’s disease from control population.

## Discussion

Even though diagnosis of AD can be reliably acquired with CSF biomarkers and neuroimaging, the need for an easier reliable plasma biomarker prompted the research on plasma clusterin. In Alzheimer’s disease, clusterin was found to be a risk factor in many genome wide association studies. [8–10] However the role of Clusterin or Apolipoprotein J in Alzheimer’s disease is still inconclusive. The proposed hypothesis for the role of clusterin in AD pathogenesis is through amyloid dependent and amyloid independent pathways.[11] Amyloid dependent pathways include the role of clusterin on Aβ aggregation and clearance, affecting the onset of Aβ deposition. Amyloid independent pathways include involvement in neuroinflammation, brain cholesterol and lipid metabolism and neuronal apoptosis. Genetic polymorphism and epigenetic mechanisms have been implicated in modulating the effect of clusterin in Alzheimer’s disease pathogenesis.[8–10]

In the study by Thambisetty et al plasma clusterin was similar between controls and patients.[5] Schrijvers et al has shown that patients had a higher plasma clusterin levels with respect to controls.[6] The hypothesis put forward in these studies was that of increased clusterin being a protective mechanism for increased amyloid clearance. In our study all the patient subgroups (MCI-AD, AD, Alzheimer pathology, MCI-VaSC, VaD and vascular pathology) had a significantly lower value of clusterin than the control samples. Plasma clusterin levels and severity of Alzheimer’s disease had no significant correlation.

Other studies have failed to show any distinction in plasma clusterin among controls and patients.[5,7] Silajdzic et al did not find any disparity in plasma clusterin values among controls, alzheimer or other dementia patients. In their study, there was a inverse correlation between clusterin values and cognitive performance (MMSE).[7]

Our results are supported by many studies which showed that various risk allele of clusterin SNP were associated with lower plasma clusterin levels. Schürmann B et al. showed that clusterin SNP risk allele rs11136000 was significantly related to lower plasma clusterin values in an allele-dose dependent manner with genotype distribution of major allele frequency in AD (0.66) and controls(0.63).[12] Xing YY et al also identified that rs9331888 allele was related to a decreased plasma clusterin values.[13] Both Schurman et al and Xing et al have shown that particular SNP risk alleles(in allele dose dependent manner) are associated with lower plasma clusterin. But in these studies either plasma clusterin is same in both groups (Schurmann et al) or raised(Xing et al). Several studies revealed that CLU/rs11136000 SNP is associated with increased CLU expression but decreased AD risk.[14–17] IJsselstijn et al showed that in presymptomatic AD patients, plasma clusterin was not elevated.[18] These results are in contradiction to the hypothesis that increased plasma clusterin is a protective mechanism.

Schrijvers et al showed that plasma clusterin was not only associated with prevalent AD, but also with all cause dementia and vascular dementia.[6] Our study showed that plasma clusterin was lower in vascular subgroup also. Plasma Clusterin thus may not be useful in differentiating between Alzheimer and Vascular dementia.

The mechanism of lower plasma clusterin is still inconclusive. Various GWAS have shown that many SNPs are associated with lower plasma clusterin along with higher risk of AD. [8–10] Higher plasma clusterin levels have shown to be associated with better cognitive performance.[7] These findings have been confirmed in our study. We hypothesize that lower clusterin values in Alzheimer’s disease may be interfering with the clearance of amyloid and inhibition of amyloid formation. Hence the amyloid cascade may be accelerated by the low clusterin levels. The low plasma clusterin may be the cause rather than the effect while accepting the known limitation that plasma values may not be representative of CSF values. The mechanism of low clusterin in vascular dementia is inconclusive. In the era of the emerging concept of mixed dementia, considering the function of clusterin in neuroinflammation and brain lipid metabolism, clusterin may have a role in the common intersecting pathways of mixed or vascular dementia. We cannot comment further since the sample size of vascular dementia was low.

Even though plasma clusterin was lower in Alzheimer subgroup compared to control group, the sensitivity and specificity of plasma clusterin was low for any clinical significance. Our study adds to the literature on plasma clusterin and showing for first time, a lower value of clusterin in Alzheimer’s disease. This questions the hypothesis of higher clusterin values being a reliable biomarker of AD especially when the sensitivity is low.

### Limitations

The sample size of the study was small as this was a pilot study. The subgroup analysis done in our study cannot be relied too much since the numbers in each group were small. A larger study on plasma clusterin in Alzheimer’s disease is currently ongoing in our institute. We did not have a histopathologically confirmed diagnosis of Alzheimer’s disease but the use of CSF biomarkers and FDG PET made the diagnosis reasonable enough. The possibility of Alzheimer pathology in vascular dementia (mixed dementia) cannot be ruled out completely even though we used FDG PET and CSF. biomarkers.

## Conclusion

Our pilot study shows that plasma clusterin is lower in Alzheimer’s disease with respect to control population. Plasma clusterin levels and severity of Alzheimer’s disease had no significant correlation. There was no difference in plasma clusterin between Alzheimer’s disease and Vascular Dementia. The sensitivity and specificity of plasma clusterin is low for any use in clinical practice. More studies are required to ascertain the utility of plasma clusterin as a biomarker in Alzheimer’s disease.

## Supporting Information

S1 File Table A: Inclusion and exclusion criteria for study and control population. Table B: Comparison of Plasma Clusterin Levels between various subgroups and controls. Table C: Comparison of plasma clusterin among Alzheimer and vascular subgroups. Table D: Baseline characteristics of Alzheimer, Vascular and Control group.
(DOC)
