# Supplementary material for: Role of Plasma Clusterin in Alzheimer’s Disease—A Pilot Study in a Tertiary Hospital in Northern India
Source: PLoS One. 2016 Nov 18;11(11):e0166369. doi: 10.1371/journal.pone.0166369 (PMC5115728; doi:10.1371/journal.pone.0166369)
Supplement: S1 File — Table A: Inclusion and exclusion criteria for study and control population. Table B: Comparison of Plasma Clusterin Levels between various subgroups and controls. Table C: Comparison of plasma clusterin among Alzheimer and vascular subgroups. Table D: Baseline characteristics of Alzheimer, Vascular and Control group. (DOC) [file pone.0166369.s001.doc]

Table A in S1 file: Inclusion and exclusion criteria for study and control population

| Inclusion Criteria of study population |
| --- |
| - Age > 50 yrs - DSM IV Criteria for Dementia - Diagnostic criteria for MCI, AD (Dubois’s criteria), VaD (DSM IV TR) - Study informant available - Adequate vision and hearing for neuropsychological testing - Normal appropriate laboratory tests - All subjects and study informants signed written consent |
| Exclusion Criteria of study population |
| - Depression - CNS infection or focal lesions of clinical significance on CT or MRI scans - Medical diseases or psychiatric disorders that could interfere with study participation |
| Inclusion Criteria of Control population |
| - Absence of memory complaints or any other cognitive symptoms - MMSE 30/30 with normal activity of daily living - Preservation of general cognitive functioning - No active neurological or psychiatric disease |

Table B in S1 file : Comparison of Plasma Clusterin Levels between various subgroups and controls

| Subgroups | | p value |
| --- | --- | --- |
| MCI AD (n=7) | Controls (n=19) | 0.004 |
| AD (n=7) | Controls (n=19) | 0.005 |
| Alzheimer group (n=34) | Controls (n=19) | 0.001 |
| MCI-VaSC (n=4) | Controls (n=19) | 0.009 |
| VaD (n=8) | Controls (n=19) | 0.001 |
| Vascular group (n=12) | Controls (n=19) | 0.0001 |

Table C in S1 file: Comparison of plasma clusterin among Alzheimer and vascular subgroups

| Parameter | Groups | | p value |
| --- | --- | --- | --- |
| Plasma clusterin | MCI AD (n=7) | MCI VaSC(n=4) | 0.850 |
|  | AD (n=27) | VaD (n=8) | 0.188 |
|  | Alzheimer’s pathology (n=34) | Vascular pathology (n=12) | 0.154 |
|  | Mild AD (n=7) | Moderate AD (n=15) | 0.275 |
|  | Mild AD (n=7) | Severe AD (n=5) | 0.167 |
|  | Moderate AD (n=15) | Severe AD (n=5) | 0.359 |

Table D in S1 file: Baseline characteristics of Alzheimer, Vascular and Control group

|  | Alzheimer | Vascular | CSF Control | Plasma Control |
| --- | --- | --- | --- | --- |
| No .of patients | 52 | 16 | 16 | 19 |
| Median age (yrs) | 67 | 60 | 62 | 65 |
| Inter-Quartile range | 61-73.75 | 55-68.25 | 56.5-65 | 60-69 |
| Male (No.) | 33 | 14 | 10 | 5 |
| Education  (No.) Illiterate | 9 | 2 | 4 | 5 |
| School (No.) | 32 | 12 | 10 | 10 |
| College (No.) | 11 | 2 | 2 | 4 |
| HTN (No.) | 18 | 14 | 8 | 8 |
| DM (No.) | 12 | 6 | 5 | 7 |
| CAD (No.) | 5 | 3 | 1 | 2 |
| DYSLIP (No.) | 6 | 12 | 8 | 12 |
| Median duration of disease (months) | 18 | 18 |  |  |
| MMSE (Median) | 18.5 | 19.5 | 30 | 30 |
| QOL (Patient) Median | 35 | 25 |  |  |
| QOL (Relative) Median | 32 | 26 |  |  |
| Median Verbal Fluency (Animals) | 7 | 7 |  |  |
| Median Verbal Fluency (COWA) | 3.8 | 3 |  |  |
| Median ADCS score | 55 | 38 |  |  |
| Median ADAS score | 13.35 | 8.6 |  |  |
| Median CDR score | 1 | 1 |  |  |
